# The impacts of energy and protein provision on anthropometric changes in infants with critical congenital heart disease

**DOI:** 10.1002/jpn3.70514

**Published:** 2026-07-23

**Authors:** Colin R. Tang‐Whitmore, Cydney Coleman, Brittany Faanes, Laura Gearman, Brian F. Joy, Stacie Knutson, Benjamin W. Langworthy, Katie M. Pfister, Catherine Larson‐Nath

**Affiliations:** ^1^ Department of Pediatrics University of Minnesota Minneapolis Minnesota USA; ^2^ Department of Nutrition MHealth Fairview Masonic Children's Hospital Minneapolis Minnesota USA; ^3^ Department of Biostatistics and Health Data Science University of Minnesota Minneapolis Minnesota USA

**Keywords:** anthropometrics, critical illness, enteral nutrition, growth, pediatric cardiology

## Abstract

**Objectives:**

Malnutrition in patients with critical congenital heart disease (CCHD) is associated with worse outcomes, however, feeding challenges, and changes in metabolic demands obscure the relationship between nutrient intake and growth in this patient population. We examined the relationship between nutrition provision (caloric and protein intake) and anthropometric indices (weight‐for‐age *z*‐score [WAZ], length‐for‐age *z*‐score [LAZ], and occipital‐frontal‐circumference‐for‐age [OAZ]).

**Methods:**

A single‐center prospective cohort study of infants admitted to cardiac intensive care at a tertiary care children's hospital with CCHD between January 2020 and August 2023. Changes in weekly anthropometric measures were modeled with weekly protein and energy intake using a generalized estimating equation model, controlling for relevant factors. The effects of meeting protein (at least 2 g/kg/day) and energy (at least 120 kcal/kg/day) goals on anthropometric *z*‐scores were also assessed, with a significance threshold of *p* < 0.05 and no corrections for multiple comparisons.

**Results:**

Thirty‐six patients were enrolled (18 male), including eight with single ventricle and 28 with biventricular physiology. Increased energy and protein intake were associated with greater expected improvements in growth parameters. Among patients who met the daily caloric goal of at least 120 kcal/kg, there was a significant increase in change in LAZ. Meeting the protein goal of at least 2 g/kg was not associated with changes in WAZ, LAZ, and OAZ.

**Conclusion:**

Increased energy and protein provision is associated with greater change in all anthropometric indices in neonates with CCHD demonstrating the importance of nutrition provision in this population.

## Abstract (highlights)

**What is Known:**

- Infants with critical congenital heart disease (CCHD) are at high risk for malnutrition and poor growth during hospitalization.
- Nutritional provision in this population is challenging due to fluid restrictions, feeding intolerance, and increased metabolic demands.
- Evidence linking actual caloric and protein intake to short‐term growth outcomes in neonates with CCHD is limited.

**What is New:**

- Higher weekly energy and protein intake were associated with improved weight‐, length‐, and head circumference—for‐age *z*‐scores during hospitalization.
- Protein intake was particularly associated with growth in the preoperative period, while energy intake supported linear growth across hospitalization.

## Graphical abstract

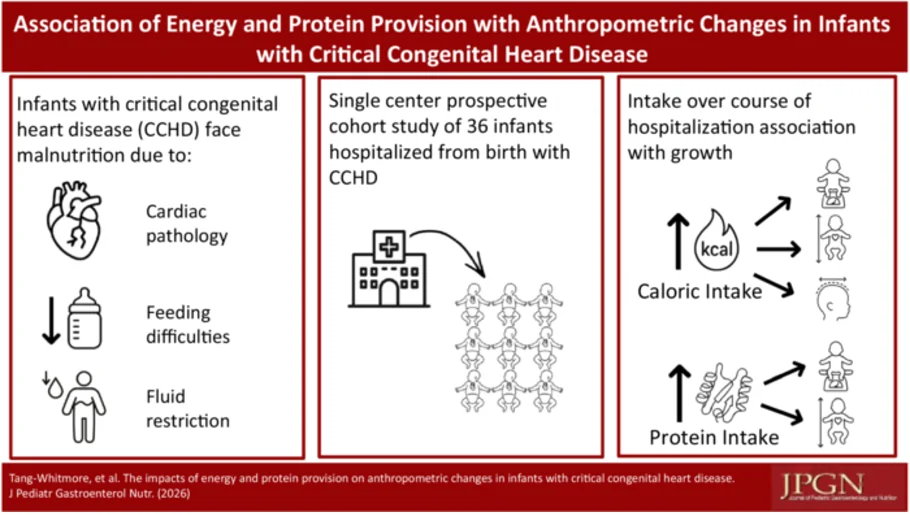

## 1 INTRODUCTION

Critical congenital heart disease (CCHD) affects approximately 20 per 10,000 live births and is associated with significant morbidity and mortality, often requiring surgical intervention within the first year of life.^1^ Malnutrition is highly prevalent in this population, with rates reported as high as 64%, and is linked to poor growth, long‐term developmental consequences, and adverse clinical outcomes.^2^, ^3^, ^4^, ^5^, ^6^, ^7^, ^8^, ^9^, ^10^, ^11^, ^12^, ^13^, ^14^ In infants with CCHD, malnutrition arises from an imbalance between nutrient intake and requirements.^15^, ^16^, ^17^, ^18^, ^19^, ^20^ During hospitalization, inadequate intake—driven by fluid restrictions, interruptions in feeding, and transiently increased postoperative energy demands—contributes to cumulative deficits in energy, protein, and micronutrients, which in turn can impair growth, increase risk of infection, prolonged hospital stay, and elevate mortality risk.^4^, ^6^, ^15^, ^16^, ^17^, ^18^, ^19^, ^20^

Although malnutrition is a potentially modifiable risk factor, providing adequate nutrition is challenging due to feeding intolerance, delayed development of oral feeding skills, fluid limitations, and increased metabolic demands related to cardiac pathology and surgery.^2^, ^4^, ^10^, ^21^, ^22^, ^23^, ^24^, ^25^, ^26^ While indirect calorimetry is considered the gold standard for assessing energy requirements in this population, it is often not feasible in routine clinical practice.^8^ As a result, nutrition is frequently guided by standard goal values for energy and protein, despite their inherent simplification of complex and often dynamic needs.

Despite these challenges, few studies have directly examined the relationship between caloric and protein intake and growth in infants with CCHD. Our prospective cohort study aimed to evaluate the association between energy and protein provision, as delivered in usual clinical care, and changes in anthropometric indices over the course of hospitalization in this population.

## 2 METHODS

### 2.1 Ethics statement

This study was approved by the University of Minnesota Institutional Review Board (IRB #00006682). Written informed consent was obtained from the parents or legal guardians of all participating neonates. All study procedures adhered to institutional ethical standards and the principles of the Declaration of Helsinki.

### 2.2 Study design and participants

This was a single‐center prospective cohort study of infants admitted with CCHD between January 2020 and August 2023. Any patient initially hospitalized within the first month of life at our institution with CCHD (including coarctation of the aorta, double‐outlet right ventricle, transposition of the great arteries, Ebstein anomaly, hypoplastic left heart syndrome, interrupted aortic arch, pulmonary atresia, total anomalous pulmonary venous connection, tetralogy of Fallot, tricuspid atresia, and truncus arteriosus) was eligible for inclusion. Infants were enrolled once informed consent was obtained from a parent or legal guardian, all within the first 4 days of life. Participants were identified through daily screening of the inpatient cardiology census in the electronic health record by research staff. No infants were excluded from participation. Eight participants had a documented genetic diagnosis. During hospitalization, anthropometric measures were collected at birth, weekly thereafter, and at discharge. Weekly average energy and protein intake were recorded over the same intervals.

### 2.3 Anthropometric data

Anthropometric measures were obtained per unit protocol, including daily weights and weekly lengths and occipital‐frontal circumferences (OFCs). Weights were measured to the nearest 0.001 kg using a portable Welch Allyn Scale‐Tronix 4802D (Skaneateles Falls, NY) or, for unstable patients, an integrated bed scale. Lengths were recorded to the nearest 0.1 cm using a Seca 417 length board (Hamburg, Germany), and OFCs to the nearest 0.01 cm using a non‐stretchable measuring tape.

Weight‐for‐length was calculated, and *z*‐scores for weight (WAZ), length (LAZ), OFC (OAZ), and weight‐for‐length were determined. World Health Organization (WHO) growth standards were used for infants ≥37 weeks corrected age, and Fenton growth charts were used for infants <37 weeks corrected age.

### 2.4 Statistical analysis

Descriptive statistics including median and interquartile ranges (IQR) for continuous variables and frequency counts for categorical variables were used to describe the patient population. The relationship between changes in weekly anthropometric measures and weekly protein and energy intake were assessed for the whole hospitalization as well as pre‐ and postoperative period. The weekly protein and energy intake was used to predict changes in anthropometric measures for the following week. Preoperative period was defined as birth until surgery and the postoperative period was post‐surgery to discharge. A generalized estimating equation (GEE) model with an AR1 correlation structure and identity link was utilized model change in anthropometric *z*‐scores. We included the number of ventricles, gestational age, and admission PRISM score as potential confounders in the GEE models. A *p*‐value < 0.05 was considered statistically significant. No adjustments were made for multiple comparisons, and as such results should be interpreted as exploratory, rather than confirming pre‐specified hypotheses. In addition, we looked at the effects of a binary indicator for whether patients met a protein goal of 2 g/kg per day and an energy goal of 120 kcal/kg per day during a given week on change in anthropometric z‐scores. We used the same modeling framework and controlled for the same potential confounders as when we used protein and caloric intake as continuous predictor variables. These goals were chosen based on the recommendations from the Neonatal Cardiac Care Collaborative (NeoC3).^8^

## 3 RESULTS

Thirty‐six patients were enrolled (18 male), 8 had single ventricle physiology, and 28 had cardiac lesions that underwent biventricular repairs (Table 1). The median gestational age was 39 (37.11, 39.32) weeks. Other demographics are reported in Table 1.

**Table 1:** Patient characteristics.

	*N*	Median (IQR)
Sex		
Female	18	
Male	18	
Ventricle physiology		
Single	8	
Biventricular	28	
Race/Ethnicity		
Asian	2	
Black or African American	2	
Native American	1	
White	26	
Hispanic or Latino	3	
Prefer not to answer	2	
Gestational age (weeks)	36	39 (37.11, 39.32)
Age at discharge (days)	36	28 (22, 38)
Admission PRISM score	36	5 (0,8)
Birth anthropometric measures (*z*‐score)		
Weight	36	−0.16 (−1.10, 0.56)
Length	36	−0.73 (−1.47, 0.79)
Occipital frontal circumference	36	−0.36 (−1.40, −0.11)

*Note*: The PRISM score is a validated severity‐of‐illness measure based on physiologic and laboratory variables obtained within the first 24 h of admission, with higher scores indicating greater severity and risk of mortality. Abbreviations: IQR, interquartile range; PRISM, pediatric risk of mortality.

The change in anthropometric *z*‐scores over the course of hospitalization was negative for all measures (Table 2). Across hospitalization, increased protein and energy intake were independently associated with increases in WAZ and LAZ and energy intake was associated with increase in OAZ. These associations remained after adjusting for biventricular physiology, PRISM score, and gestational age (Tables 3 and 4). In the energy intake model, biventricular physiology was associated with greater positive changes in LAZ and OAZ, and higher PRISM scores on admission were associated with greater increases in WAZ (Table 3). Greater gestational age was linked to larger increases in OAZ (Table 3). In the protein intake model, gestational age was associated with greater changes in WAZ and OAZ (Table 4).

**Table 2:** Change in *z*‐score over duration of hospitalization.

Anthropometric measure	*N*	Median (IQR)
Weight	36	−1.03 (−1.81, −0.50)
Length	36	−0.52 (−1.06, 0.12)
Occipital frontal circumference	36	−0.46 (−1.02, −0.22)

Abbreviation: IQR, interquartile range.

**Table 3:** Relationship between energy intake and change in anthropometric *z*‐score for the following week.

Characteristic	Beta^a^	95% CI	*p*‐value
Outcome: Weight *z*‐score (*N* = 60)			
Total calories (per 10 kcal/kg)	**0.06**	**0.00–0.14**	**0.037**
Biventricular versus single ventricle physiology	0.18	−0.28 to 0.63	0.40
Gestational age (weeks)	0.03	−0.02 to 0.09	0.20
Admission PRISM score	0.01	−0.01 to 0.04	0.20
Outcome: Length *z*‐score (*N* = 51)			
Total calories (per 10 kcal/kg)	**0.15**	**0.09–0.21**	**<0.001**
Biventricular versus single ventricle physiology	**0.61**	**0.25–0.97**	**<0.001**
Gestational age (weeks)	0.04	−0.04 to 0.12	0.30
Admission PRISM score	0.03	−0.01 to 0.08	0.13
Outcome: OFC *z*‐score (*N* = 55)			
Total calories (per 10 kcal/kg)	**0.15**	**0.05–0.22**	**0.003**
Biventricular versus single ventricle physiology	**0.65**	**0.24–0.90**	**<0.001**
Gestational age (weeks)	**0.09**	**0.04–0.14**	**<0.001**
Admission PRISM score	**0.03**	**0.00–0.06**	**0.037**

*Note*: Bolded values indicate statistical significance (*p* < 0.05).

Abbreviations: CI, confidence interval; OFC, occipital frontal circumference; PRISM, pediatric risk of mortality.

^a^ Beta represents the estimated change in *z*‐score per unit increase in energy intake.

**Table 4:** Relationship between protein intake and change in anthropometric *z*‐score for the following week.

Characteristic	Beta^a^	95% CI	*p*‐value
Outcome: Weight *z*‐score (*N* = 60)			
Total protein (per 1 g/kg)	**0.29**	**0.14–0.44**	**<0.001**
Biventricular versus single ventricle physiology	0.10	−0.37 to 0.58	0.7
Gestational age (weeks)	**0.07**	**0.02–0.12**	**0.005**
Admission PRISM score	0.01	−0.01 to 0.03	0.5
Outcome: Length *z*‐score (*N* = 52)			
Total protein (per 1 g/kg)	**0.23**	**0.02–0.43**	**0.028**
Biventricular versus single ventricle physiology	0.34	−0.12 to 0.79	0.14
Gestational age (weeks)	0.01	−0.10 to 0.12	0.9
Admission PRISM score	0.01	−0.05 to 0.07	0.7
Outcome: OFC *z*‐score (*N* = 56)			
Total protein (per 1 g/kg)	0.20	−0.03 to 0.43	0.088
Biventricular versus single ventricle physiology	0.41	−0.03 to 0.85	0.065
Gestational age (weeks)	**0.07**	**0.00–0.14**	**0.046**
Admission PRISM score	0.01	−0.02 to 0.05	0.5

*Note*: Bolded values indicate statistical significance (*p* < 0.05).

Abbreviations: CI, confidence interval; OFC, occipital frontal circumference; PRISM, pediatric risk of mortality.

^a^ Beta represents the estimated change in *z*‐score per unit increase in protein intake.

### 3.1 Pre‐ versus post‐operative period

In the pre‐operative period, each increase of 10 kcal/kg in caloric consumption led to increases in the expected weight *z*‐score, (*β* = 0.09, 95% confidence interval [CI] 0.02, 0.15; *p* = 0.009), OFC *z*‐score (*β* = 0.13, 95% CI 0.04, 0.23; *p* = 0.007), and weight for length *z*‐score (*β* = 0.52, 95% CI 0.42, 0.61; *p* < 0.001). There were also significant increases in the expected weight *z*‐score (*β* = 0.27, 95% CI 0.12, 0.41; *p* < 0.001), length *z*‐score (*β* = 0.20, 95% CI 0.05, 0.35; *p* = 0.008), and OFC *z*‐score (*β* = 0.51, 95% CI 0.14, 0.88; *p* = 0.007) for each g/kg increase in protein provision. In the post‐operative period, there were significant increases in the expected length *z*‐score (*β* = 0.17, 95% CI 0.12, 0.22; *p* < 0.001) change for each 10 kcal/kg increase in caloric consumption.

### 3.2 Meeting calorie goal

Among patients who met the daily caloric intake goal of at least 120 kcal/kg, meeting this target was associated with a significant increase in change in length‐for‐age *z*‐score (*β* = 0.42, 95% CI: 0.23–0.61; *p* < 0.001). No significant associations were observed between caloric intake and changes in WAZ or OAZ (see Supporting Information S1: Table S1). However, after adjusting for ventricle physiology, greater change in OFC *z*‐score was observed among patients meeting the caloric goal (*β* = 0.42, 95% CI: 0.01–0.83; *p* = 0.047). The caloric goal was met on only nine occasions.

### 3.3 Meeting protein goal

Among patients who met the daily protein intake goal of at least 2 g/kg, there were no significant changes in WAZ, LAZ, or OAZ (see Supporting Information S1: Table S2). However, after adjusting for ventricle physiology, meeting the protein goal was associated with a significant increase in change in OAZ (*β* = 0.46, 95% CI: 0.01–0.90; *p* = 0.046).

## 4 DISCUSSION

Neonates with CCHD experience significant stress during a critical period for growth and development. They are at high risk for malnutrition due to the impacts of their cardiac pathologies, increased resting energy expenditure, and significant surgical intervention often requiring cardiopulmonary bypass, which is compounded by poor tolerance for oral and enteral feeding. Cumulative nutritional deficits in the neonatal period can have negative impacts on their growth and development long term; therefore, there has been increased attention on mitigating nutritional deficits during this period. While indirect calorimetry remains the gold standard for assessing energy requirements in this population, it is not routinely feasible or clinically practical in most neonatal intensive care settings.^8^ Consequently, employing standardized caloric and protein targets represents a necessary and pragmatic approach to guide nutritional support, despite its inherent simplifications. There remains a question, however, whether malnutrition in this population is due to inadequate intake or an inability to utilize nutrition provided. Our prospective cohort demonstrated a decrease in all anthropometric measures over the course of hospitalization, consistent with previous reports.^27^ However, there was a positive relationship between weeks with increased caloric and protein intake and greater changes in anthropometric z‐scores.

Specific nutritional guidelines for neonates and infants with CCHD remain limited. Exact protein requirements for this population are unclear; however, it is well recognized that infants with critical illness often experience protein deficiency and muscle wasting.^28^ Although the NeoC3 guidelines do not provide firm recommendations, they suggest that protein requirements likely exceed 1.5 g/kg/day and that energy needs are often higher, though these may fluctuate throughout hospitalization.^8^ Current ASPEN guidelines for term neonates and infants recommend 2–3 g/kg/day of protein and approximately 120 kcal/kg/day, which informed our decision to evaluate 2–3 g/kg/day of protein and 120–140 kcal/kg/day of energy intake in our study.^29^ Importantly, high protein supplementation and surplus intake are not recommended due to insufficient evidence and concerns regarding nephrotoxicity.^30^, ^31^ Supporting the importance of adequate protein intake, Botrán et al. demonstrated improved outcomes among critically ill children receiving 2.6 g/kg/day compared with 1.5 g/kg/day, although this study was conducted in a broader PICU population ranging from 1 month to 16 years of age.^30^

Meeting the caloric intake goals for neonates with CCHD is critical for supporting growth and development, yet our study reveals that the majority of the patients in the cohort did not achieve the recommended energy intake requirements. NeoC3 provides evidence‐based guidelines for nutritional support for neonates and infants with CHD.^8^ Healthy full‐term neonates require 90–120 kcal/kg/day, however, neonates with CCHD have been shown to have up to 35% greater total energy expenditure than age‐matched controls in meta‐analysis of cohort studies.^32^, ^33^, ^34^ This population requires between 140 and 150 kcal/kg/day to maintain linear growth and increase subcutaneous fat and muscle, especially in the preoperative period.^33^ Our study aimed to assess the association between meeting the caloric intake goal and growth during hospitalization, however the analysis was not feasible because only 2 of the 36 patients in the cohort achieved the intake goal. Despite the inability to complete the analysis, the finding that the majority of the cohort did not meet the caloric intake goal is noteworthy. Indeed, other tertiary centers have encountered similar challenges. A recent retrospective chart review identified hemodynamic instability and invasive ventilation as barriers to meeting the optimal nutritional intake guidelines outlined by ASPEN.^35^ This highlights the significant challenges with nutrition provision faced by this population and the need for targeted interventions to address their nutritional needs.

In our cohort, meeting protein goals between 2 and 3 g/kg/day was associated with significantly increased LAZ and HAZ in the preoperative period, while meeting calorie targets of 120–140 kcal/kg/day predicted a significant increase in LAZ across the entire study period. Notably, the impact of protein intake was only significant in the preoperative period, suggesting that protein needs may differ throughout hospitalization and may be particularly critical during periods of acute physiologic stress consistent with previous reports.^8^, ^30^ This underscores the potential value of flexible, patient‐tailored nutritional strategies to optimize growth when infants are most vulnerable, such as prior to surgical intervention. Similarly, the association between adequate calorie intake and improved LAZ across the entire hospitalization emphasizes the importance of consistent energy provision, which may support linear growth and better long‐term developmental outcomes. Together, these results reinforce the clinical significance of achieving recommended protein and calorie intake goals and suggest that flexible, patient‐tailored approaches may be essential for optimizing growth in neonates with CCHD.

Energy requirements change in neonates with CCHD through the course of the disease with higher energy requirements in the unrepaired, pre‐operative state.^34^, ^36^ Our study found significant expected change in WAZ, LAZ, and OAZ for increased energy and protein intake, which supports prior findings that meeting the higher energy requirements of this population promotes growth even in the unrepaired state. The postoperative state may be either hyper‐ or hypometabolic and often varies in duration.^31^, ^37^, ^38^, ^39^, ^40^, ^41^ Changes in energy requirements in the postoperative state typically resolved 1 week following cardiac surgery.^36^ In the post‐operative period in our cohort, there was a significant increase in LAZ with increased energy intake. This is possibly due to a delay in linear growth from nutrition provided in the pre‐operative, unrepaired period.

Our study revealed that infants with biventricular physiology demonstrated more pronounced growth changes (as measured by LAZ and OAZ) in response to energy intake. This may be attributed to the more efficient circulatory systems in these infants, which likely support better nutrient delivery to tissues. Greater gestational age was associated with more significant changes in LAZ which demonstrates the unique nutritional needs of premature infants.

While this study provides valuable insights about the association between nutrition provision and anthropometric change, there are limitations that should be acknowledged. First, the sample size of the cohort is small, which limits our ability to assess whether there are more critical periods during hospitalization where increased nutrition intake may be more beneficial (pre‐operative vs. peri‐operative vs. post‐operative period). In addition, due to the small size, more specific analysis of differences in growth between patients with differing cardiac pathologies was not possible, and the cohort, though all meeting criteria for CCHD, remains relatively heterogeneous. We were also not able to complete the analysis for changes in weight‐for‐length *z*‐score due to limited length measurements at the time of discharge. Next, our intake data were broken down into units of protein and calories and do not assess associations between the type of nutrition provided and growth. Comparison between types of nutrition, including human milk, formula, and parenteral nutrition, would provide additional nuance on how the type and delivery of nutrition impact growth. Lastly, while our institution has standardized protocols for pre‐operative feeding in all ductal‐dependent neonates and a post‐operative feeding protocol for all single ventricle patients, variation in CVICU nutritional advancement protocols may have introduced variability in how and when diets were adjusted and advanced. This may have confounded our analysis by influencing the consistency and timing of nutritional interventions, making it difficult to assess nutrition provision's impact on growth outcomes across the cohort.

Despite these limitations, several strengths enhance confidence in our findings. The prospective study design minimizes selection bias, as participants were identified and enrolled at birth and followed longitudinally throughout their hospitalization, ensuring nearly complete follow‐up. Furthermore, anthropometric measurements were performed using standardized protocols and appropriate techniques, reducing the risk of measurement error and related bias.

## 5 CONCLUSION

In conclusion, our findings underscore the importance of ensuring adequate caloric and protein intake in infants with CCHD during hospitalization. Clinical efforts to optimize oral, enteral, and parenteral nutrition, tailored to each infant's specific needs and tolerances, may support growth in this population and improve outcomes.

## CONFLICT OF INTEREST STATEMENT

The authors declare no conflicts of interest.

## Supporting information

Supporting information.
