# Supplementary material for: The impacts of energy and protein provision on anthropometric changes in infants with critical congenital heart disease
Source: J Pediatr Gastroenterol Nutr. 2026 Jul 23;83(4):737–43. doi: 10.1002/jpn3.70514 (PMC13646035; doi:10.1002/jpn3.70514)
Supplement: Supplementary file 1 [file JPN3-83-737-s001.docx]

Supplementary Table S1: Generalized estimating equation (GEE) analysis with an AR(1) correlation structure evaluating the relationship between meeting the daily total caloric intake goal (≥120 kcal/kg/day) and change in anthropometric z-score for the subsequent week over the course of hospitalization.

| **Characteristic** | **Beta*** | **95% CI** | **p-value** |
| --- | --- | --- | --- |
| **Outcome: Weight z-score** (N = 60) |  |  |  |
| Met Average Daily Calorie Goal | 0.03 | -0.08, 0.15 | 0.6 |
| Biventricular vs single ventricle physiology | 0.10 | -0.37, 0.56 | 0.7 |
| Gestational Age (weeks) | 0.00 | -0.05, 0.05 | 0.9 |
| Admission PRISM Score | 0.01 | -0.02 , 0.03 | 0.6 |
| **Outcome: Length z-score** (N = 51) |  |  |  |
| Met Average Daily Calorie Goal | **0.42** | **0.23, 0.61** | **<0.001** |
| Biventricular vs single ventricle physiology | 0.34 | -0.13, 0.81 | 0.2 |
| Gestational Age (weeks) | -0.03 | -0.12, 0.07 | 0.6 |
| Admission PRISM Score | 0.02 | -0.05, 0.08 | 0.6 |
| **Outcome: OFC z-score** (N = 56) |  |  |  |
| Met Average Daily Calorie Goal | 0.61 | -0.61, 1.8 | 0.3 |
| Biventricular vs single ventricle physiology | **0.42** | **0.01, 0.83** | **0.047** |
| Gestational Age (weeks) | 0.04 | -0.03 to 0.11 | 0.2 |
| Admission PRISM Score | 0.02 | -0.02 to 0.06 | 0.3 |

*Beta represents the estimated change in z-score per unit increase in energy intake. Bolded values indicate statistical significance (p < 0.05). CI = Confidence interval; PRISM = Pediatric Risk of Mortality.

Supplementary Table S2: Generalized estimating equation (GEE) analysis with an AR(1) correlation structure evaluating the relationship between meeting the daily total protein goal (≥2 g/kg) and change in anthropometric z-score for the subsequent week over the course of hospitalization, adjusted for cardiac physiology (biventricular vs. single ventricle), gestational age, and admission PRISM score.

| **Characteristic** | **Beta*** | **95% CI** | **p-value** |
| --- | --- | --- | --- |
| **Outcome: Weight z-score** (N = 61) |  |  |  |
| Met Average Daily Protein Goal | 0.01 | -0.30, 0.33 | >0.90 |
| Biventricular vs single ventricle physiology | 0.13 | -0.33, 0.59 | 0.60 |
| Gestational Age (weeks) | 0.00 | -0.05, 0.05 | >0.9 |
| Admission PRISM Score | 0.01 | -0.02 , 0.03 | 0.70 |
| **Outcome: Length z-score** (N = 52) |  |  |  |
| Met Average Daily Protein Goal | 0.05 | -0.24, 0.34 | 0.80 |
| Biventricular vs single ventricle physiology | 0.35 | -0.12, 0.83 | 0.14 |
| Gestational Age (weeks) | -0.04 | -0.14, 0.05 | 0.40 |
| Admission PRISM Score | 0.01 | -0.05, 0.07 | 0.80 |
| **Outcome: OFC z-score** (N = 56) |  |  |  |
| Met Average Daily Protein Goal | 0.14 | -0.22, 0.50 | 0.40 |
| Biventricular vs single ventricle physiology | **0.46** | **0.01, 0.90** | **0.046** |
| Gestational Age (weeks) | 0.02 | 0.00 to 0.13 | 0.08 |
| Admission PRISM Score | 0.01 | -0.03 to 0.05 | 0.70 |

*Beta represents the estimated change in z-score per unit increase in energy intake. Bolded values indicate statistical significance (p < 0.05). CI = Confidence interval; PRISM = Pediatric Risk of Mortality.
